# Alcohol Drinking and Health in Ageing: A Global Scale Analysis of Older Individual Data through the Harmonised Dataset of ATHLOS

**DOI:** 10.3390/nu12061746

**Published:** 2020-06-11

**Authors:** Stefanos Tyrovolas, Dimitris Panaretos, Christina Daskalopoulou, Iago Gine-Vazquez, Albert Sanchez Niubo, Beatriz Olaya, Martin Bobak, Martin Prince, Matthew Prina, Jose Luis Ayuso-Mateos, Francisco Felix Caballero, Esther Garcia-Esquinas, Arndt Holger, Sergei Scherbov, Warren Sanderson, Ilenia Gheno, Ilona Koupil, Jerome Bickenbach, Somnath Chatterji, Seppo Koskinen, Alberto Raggi, Andrzej Pajak, Beata Tobiasz-Adamczyk, Josep Maria Haro, Demosthenes Panagiotakos

**Affiliations:** 1 Parc Sanitari Sant Joan de Déu, Fundacion Sant Joan de Deu, 42, 08830 Sant Boi de Llobregat, Spain; i.gine@pssjd.org (I.G.-V.); albert.sanchez@pssjd.org (A.S.N.); beatriz.olaya@pssjd.org (B.O.); jmharo@pssjd.org (J.M.H.); 2 Instituto de Salud Carlos III, Centro de Investigación Biomédica en Red de Salud Mental, CIBERSAM, Monforte de Lemos 3–5, Pabellón 11, 28029 Madrid, Spain; joseluis.ayuso@uam.es; 3 Department of Nutrition and Dietetics, School of Health Science and Education, Harokopio University, 70 Eleftheriou Venizelou Ave, Attica, 176 61 Athens, Greece; dimitrispanaretos@hotmail.com (D.P.); dbpanag@hua.gr (D.P.); 4 Psychology and Neuroscience, Department of Health Service and Population Research, Institute of Psychiatry, King’s College London, London WC1E 6BT, UK; christina.daskalopoulou@kcl.ac.uk (C.D.); martin.prince@kcl.ac.uk (M.P.); matthew.prina@kcl.ac.uk (M.P.); 5 Research Department of Epidemiology and Public Health, University College London, 1–19 Torrington Place, London WC1E 7HB, UK; m.bobak@ucl.ac.uk; 6 Department of Psychiatry, Universidad Autónoma de Madrid, 28049 Madrid, Spain; 7 Hospital Universitario de La Princesa, Instituto de Investigación Sanitaria Princesa (IIS Princesa), 28006 Madrid, Spain; 8 Department of Preventive Medicine and Public Health, School of Medicine, Universidad Autónoma de Madrid, 28049 Madrid, Spain; felix.caballero@uam.es (F.F.C.); esthergge@gmail.com (E.G.-E.); 9 CIBER of Epidemiology and Public Health-CIBERESP, 28029 Madrid, Spain; 10 SPRING TECHNO GMBH & Co. KG, 28199 Bremen, Germany; h.arndt@springtechno.com; 11 World Population Program, International Institute for Applied Systems Analysis, Wittgenstein Centre for Demography and Global Human Capital, 2361 Laxenburg, Austria; sergei.scherbov@oeaw.ac.at (S.S.); warren.sanderson@stonybrook.edu (W.S.); 12 Vienna Institute of Demography, Austrian Academy of Science, 1030 Vienna, Austria; 13 International Laboratory for Demography and Human Capital, Russian Presidential Academy of National Economy and Public Administration (RANEPA), 119571 Moscow, Russia; 14 Department of Economics, Stony Brook University, Stony Brook, NY 11794, USA; 15 AGE Platform, 1150 Brussels, Belgium; ilenia.gheno@age-platform.eu; 16 Department of Public Health Sciences, Centre for Health Equity Studies, Stockholm University, 114 19 Stockholm, Sweden; ilona.koupil@su.se; 17 Department of Global Public Health, Karolinska Institutet, 171 77 Stockholm, Sweden; 18 Department of Health Sciences and Health Policy, University of Lucerne, 6002 Lucerne, Switzerland; jerome.bickenbach@paraplegie.ch; 19 Swiss Paraplegic Research, 6207 Nottwil, Switzerland; 20 Information, Evidence and Research, World Health Organization, 1202 Geneva, Switzerland; chatterjis@who.int; 21 Department of Public Health Solutions, Finnish Institute for Health and Welfare (THL), P.O. Box 30, FI-00271 Helsinki, Finland; seppo.koskinen@thl.fi; 22 Fondazione IRCCS Istituto Neurologico Carlo Besta, 20133 Milan, Italy; alberto.raggi@istituto-besta.it; 23 Department of Epidemiology and Population Studies, Faculty of Health Sciences, Jagiellonian University Medical College, 31-008 Krakow, Poland; andrzej.pajak@uj.edu.pl; 24 Department of Medical Sociology, Department of Epidemiology, Chair of Epidemiology and Preventive Medicine, Jagiellonian University Medical College, 31-008 Krakow, Poland; mytobias@cyf-kr.edu.pl

**Keywords:** alcohol drinking, health status, ageing, older adults, ATHLOS

## Abstract

We investigated the relation between alcohol drinking and healthy ageing by means of a validated health status metric, using individual data from the Ageing Trajectories of Health: Longitudinal Opportunities and Synergies (ATHLOS) project. For the purposes of this study, the ATHLOS harmonised dataset, which includes information from individuals aged 65+ in 38 countries, was analysed (*n* = 135,440). Alcohol drinking was reflected by means of three harmonised variables: alcohol drinking frequency, current and past alcohol drinker. A set of 41 self-reported health items and measured tests were used to generate a specific health metric. In the harmonised dataset, the prevalence of current drinking was 47.5% while of past drinking was 26.5%. In the pooled sample, current alcohol drinking was positively associated with better health status among older adults ((b-coef (95% CI): 1.32(0.45 to 2.19)) and past alcohol drinking was inversely related (b-coef (95% CI): −0.83 (−1.51 to −0.16)) with health status. Often alcohol consumption appeared to be beneficial only for females in all super-regions except Africa, both age group categories (65–80 years old and 80+), both age group categories, as well as among all the financial status categories (all *p* < 0.05). Regional analysis pictured diverse patterns in the association for current and past alcohol drinkers. Our results report the need for specific alcohol intake recommendations among older adults that will help them maintain a better health status throughout the ageing process.

## 1. Introduction

The relation between alcohol drinking and health remains quite complex. Alcohol drinking has been documented as a risk factor for chronic diseases and disability [1,2,3]. A variety of research studies have supported the notion that low or moderate consumption of alcohol is related to better health outcomes [4], while recent well-documented studies disagree and report the effect of “hidden systematic error” [5,6]. The Global Burden of Disease (GBD) 2016 alcohol study supported that no alcohol drinking is the only level that minimises the loss of health at the population level [7]. 

The global population is ageing with unpreceded speed, while the old (65+ years old) and oldest old (90+ years old) are now the fastest growing population segment in various regions. People 65+ may be at greater health risk due to alcohol drinking, taking into account the body´s ageing-related physiological changes (e.g., blood ethanol concentration, lower hepatic function) and alcohol intake is contraindicated with medical drug prescriptions [8,9]. Recent data indicate that, among older adults, alcohol drinking is a part of social engagement and the health risks of alcohol are not widely accepted [10]. Five years ago (2014), the World Health Organization (WHO) launched a global strategy that reported that the prevention of and reduction in the harmful use of alcohol is a priority for public health. However, to design prevention strategies and interventions targeted to older adults, we have to understand the beneficial or adverse effects of alcohol drinking for healthy ageing, something that, to date, has been missing.

Despite rapid population ageing and the inconsistent findings about the relationship between alcohol intake and various ageing-related concepts [11,12,13] (e.g., frailty, sarcopenia), there are no global epidemiological data on the effect of alcohol consumption on healthy ageing. In particular, although recently interesting population-based results on the effect of alcohol or healthy lifestyle habits on health have been reported [14,15] there is no multi-national study with individual data investigating the relationship between alcohol drinking and healthy ageing, for individuals over the age of 65, that allows for regional and temporal comparisons. 

The aim of the present study, therefore, is to evaluate the relation between alcohol drinking (drinking frequency, current and past drinking) and healthy ageing by means of a validated health status metric in 38 countries using individual data from the Ageing Trajectories of Health: Longitudinal Opportunities and Synergies (ATHLOS) project. The ATHLOS project (EU HORIZON2020–PHC-635316, http://athlosproject.eu/) is an international collaborative project involving international ageing cohort studies (17 studies). It aims to achieve a better understanding of ageing by identifying patterns of healthy ageing trajectories, determinants of those patterns, critical points in time when changes in trajectories are produced, and to propose timely clinical and public health interventions to optimise healthy ageing. 

## 2. Methods

The ATHLOS mega dataset included 411,000 participants from seventeen population-based cohort studies across the world as listed below [16]. The studies are the 10/66 Dementia Research Group Population-Based Cohort Study, the Australian Longitudinal Study of Aging (ALSA), The ATTICA Study, China Health and Retirement Longitudinal Study (CHARLS), Collaborative Research on Ageing in Europe (COURAGE), English Longitudinal Study of Ageing (ELSA), Study on Cardiovascular Health, Nutrition and Frailty in Older Adults in Spain (ENRICA), the Health, Alcohol and Psychosocial factors in Eastern Europe Study (HAPIEE), the Health 2000/2011 Survey, Health and Retirement Study (HRS), Japanese Study of Aging and Retirement (JSTAR), Korean Longitudinal Study of Ageing (KLOSA), Mexican Health and Aging Study (MHAS), Study on Global Ageing and Adult Health (SAGE), Survey of Health, Ageing and Retirement in Europe (SHARE), the Irish Longitudinal Study of Ageing (TILDA), and the Uppsala Birth Cohort Multigenerational Study (UBCoS). 

Characteristics describing each cohort are catalogued by the ATHLOS rigorous harmonisation procedure [17]. ATHLOS consortium members defined a set of variables to be generated from the harmonisation process. These were: (1) socio-demographic and economic characteristics; (2) lifestyle and health behaviours; (3) health status and functional limitations; (4) diseases; (5) death; (6) physical measures; (7) psychological measures; (8) laboratory measures; (9) social environment and life events, and (10) other administrative information [16].

All cohorts had gained approval through their local research ethics committees or institutional review board for the secondary usage of data. In addition, each cohort´s participants gave their consent to their study of origin. The ATHLOS study protocol was also approved under the data access and ethics governance requirements of the study of origin. A detailed description of the ATHLOS cohort and all the participating studies and harmonisation procedures have published elsewhere [16]. 

For the purposes of this study, we analysed information from the ATHLOS pooled sample for those 65+ years old (*n* = 135,440). As UBCoS did not include all the relevant measurements, the harmonised data from the UBCOS could not be used in this analysis.

### 2.1. Metric of Health Composition in Ageing

The estimation and validation of the health status metric was evaluated using the ELSA dataset [18], which is part of the ATHLOS mega-dataset. The metric of health, constructed according to the notion of the ‘health state’, has been proposed by the World Health Organization, and covers the intrinsic attribute of an individual that can be aggregated to the population level as well as includes domains of human functioning that reflect the real impact of health conditions on people’s lives [19]. Thus, the constructed metric of health evaluates health status conceptualised as a source of various functioning domains, ranging from the simplest to the most complex one (e.g., walking, vision, Activities of Daily Living (ADL) and Instrumental Activities of Daily Living (IADL)). Based on this methodological approach [18], 41 variables were identified in the ATHLOS mega-dataset, encompassing 35 self-reported health questions related to impairments in bodily functions (i.e., vision, hearing problems), limitations in ADL, and limitations in IADL, and another five items related to cognitive functioning (i.e., delayed recall, immediate recall) and walking speed (measured test of meters per second). The theoretical range of the health status metric was from zero to 100; higher values in the health metric score report of better health status.

### 2.2. Other Participants’ Characteristics

Basic socio-demographic characteristics such as age, gender, education, region of residence, financial status, and lifestyle characteristics, such as living alone, smoking habits and physical activity status, clinical and anthropometric characteristics such as established cardiovascular disease and body mass index (BMI) were harmonised and included in this study. Using their region of residence, individuals were grouped in four super-regions that were Europe, Asia and Oceania, Africa and North and South America. Participants’ education level, across all individual ATHLOS studies, was harmonised in a four-scale item (no education, primary, secondary, tertiary-university level) based on the International Standard Classification of Education (i.e., early childhood education, lower secondary education, short-cycle tertiary education) [20]. Financial status was assessed using wealth quintile groups, which was selected because of the variety of the populations studied, as well as the common difficulty of accessing exact financial data. Living alone was grouped in a binary variable based on the provided living condition of the older individual (alone vs. not alone). Smoking habits were harmonised in the group past or current smokers vs. never smokers. Smokers were those that reported smoking over the course of their life. Physical activity was harmonised among all cohorts in a four-scale item (inactive, low, moderate, high physically active). As inactive were classified those with no physical activity and the rest three classifications were created based on the frequency and intensity of physical activity. BMI was calculated as weight in kilograms divided by height in meters squared. Cardiovascular disease (CVD) was harmonised among all the cohorts as having an individual established heart disease (coronary heart disease, myocardial infraction, stroke, angina) assessed by a physician or not [16]. The harmonisation algorithms of the aforementioned variables can be found at https://github.com/athlosproject/athlos-project.github.io.

### 2.3. Alcohol Drinking 

Each study included in the ATHLOS repository collected the specific food and beverage intake information through their own semi-quantitative questionnaires based on the frequency of consumption (for more information please see particular studies). These questionnaires explained in detail to the interviewed individual the difference between non-alcoholic and alcoholic beverages. As an example in the COURAGE study, part of the ATHLOS dataset, alcohol drinking was evaluated with the following questions: “Have you ever consumed a drink that contains alcohol (such as beer, wine, alcohol spirits, etc.)?” with answer options ‘yes’ and ‘no’. A separate question then asked about how many drinks of any alcohol beverage the participant had consumed each day of the past week. In the ATHLOS dataset, information on alcohol drinking was homogenised among the different studies throughout two binary (current and past drinker) and one three-point scale variables (frequency of drinking). In particular, frequency of alcohol was categorised as never for those never consuming any alcoholic drink, rare for those consuming less than one alcoholic drink per week, and often for all the other frequency of consumption. As current drinkers were defined as all those that were consuming alcohol at the time of the survey, while past alcohol drinkers were defined as those persons who used to consume any alcoholic drink in the past, but that stopped any consumption by the past year (for more information, see http://athlosproject.eu/).

### 2.4. Statistical Analysis

The analysis was restricted to those aged 65 years or older because of the age-related nature of healthy ageing. Normally distributed continuous variables were presented as mean ± SD and categorical variables as frequencies. Comparisons of continuous variables between groups were performed using the independent samples t-test (for normal distribution) and the Mann–Whitney U-test (for skewed distribution). Moreover, a multiple linear regression analysis was performed in order to evaluate the association between the health status metric (dependent outcomes), alcohol drinking (frequency of alcohol drinking, past and current alcohol drinker) and other participant characteristics (i.e., age, sex, living alone education and financial status, smoking habits, physical activity, BMI and CVD—independent variables). Collinearity was tested using the Variance Inflation Factor criterion (VIF; values >4 suggested collinearity between independent variables and one of them was excluded from the model). The assumption of homoscedasticity was tested by plotting the scatter plot of standardised residuals over the predicted score values. Results from linear regression models are presented as b-coefficients (b-coef) and their 95% confidence intervals (95%CI). All reported p-values were based on two-sided tests. R software (version 3.5.3, R Foundation for Statistical Computing, Vienna, Austria) was used for all calculations.

## 3. Results

After the exclusion of those <65 years, the sample size was *n* = 135,440. The prevalence of current drinking was 47.5%, of past drinking 26.5% and while almost 51% had never consumed alcohol, 27% rarely consumed it and 22% were frequent alcohol users (data shown only in text).

Table 1 presents the baseline characteristics of the sample by alcohol drinking. Among older adults, males were more frequent alcohol drinkers than females (*p* < 0.001). Older adults with the highest education and financial status, as well as the never smokers, the more physically active and those with established CVD, reported the lowest frequency of alcohol drinking (*p* < 0.001). Current alcohol consumers were more likely to be males, with a middling level of education, low to middling financial status and smokers, (*p* < 0.001). A similar pattern was also pictured for the former alcohol drinkers.

The association between alcohol drinking and the health status metric, estimated by multivariable linear regression, is shown in Table 2. In the pooled sample, frequent (often-level) alcohol drinking was positively associated with better health status compared with no alcohol intake (b-coef (95% CI): 3.70 (2.67 to 4.73)), after adjusting for various confounders. Current alcohol drinking was positively associated with better health status among older adults ((b-coef (95% CI): 1.32 (0.45 to 2.19)), while past alcohol drinking was inversely related (b-coef (95% CI): −0.83 (−1.51 to −0.16)) with health status. The different effect of current and past alcohol drinking on the health status of older adults among different age groups was also assessed. At 85+ years old, a certain declining trend was reported between alcohol drinking (b-coef (95% CI): 3.01 (−1.60 to 7.62)) and health status while an increasing one was shown for former alcohol drinking (b-coef (95% CI): 6.15 (−0.97 to 13.2)) (data shown only in text).

**Figure 1:**
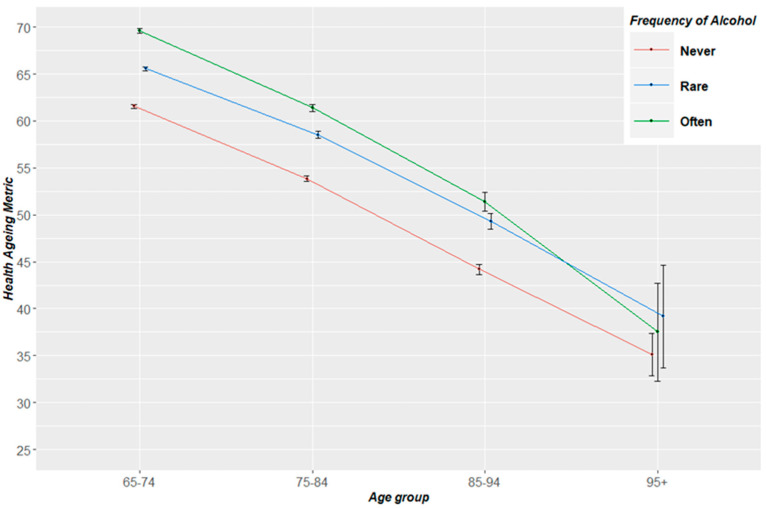
Trajectories of health status from the ages of 65 to 95+ years old, by frequency of alcohol drinking for the total ATHLOS sample. Health ageing metric ranges from 0–100; Ageing Trajectories of Health: Longitudinal Opportunities and Synergies (ATHLOS).

**Figure 2:**
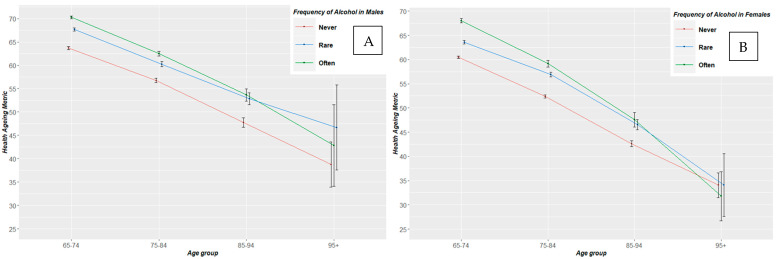
Trajectories of health status metric from the ages of 65 to 95+ years old by frequency of alcohol drinking for males (**A**) and females (**B**). Healthy ageing metric ranges from 0–100.

Patterns of health status from the ages of 65 to 95+ years old, by frequency of alcohol drinking, for the pooled sample, are shown in Figure 1. The older never drinking population was the lowest health status trajectory among all age groups. Those with frequent (often-level) alcohol consumption had a better health status trajectory between 65 and 85 years old, followed by those with rare alcohol intake. However, beyond the age group of 85–94 years old, rare or frequent alcohol converged, with rare alcohol intake picturing a better health trajectory among the population of those 95+ years old. Similar trajectories followed when this analysis was applied by gender (Figure 2). Interestingly, only females beyond age of 95 that never consumed alcohol pictured a better health trajectory than those with rare or often consumption. 

In order to evaluate age, gender, financial, and geographic patterns of alcohol drinking and the health status metric, separated clustered analysis was applied (Table 3). Often alcohol drinking was beneficial for females, in all super-regions except in African countries, both younger older adults and the octogenarians, as well as among all categories of financial status (all *p* < 0.05). The association was particularly pronounced among North and South Americans than in Asian and Oceanian populations and Europeans. Diverse patterns pictured in the association the current and past alcohol drinkers. In particular for current and past alcohol drinkers, positive and reversed relations were reported, among “younger” older adults, females as well as the poorest and richest older populations (all *p* < 0.05). Regional analysis showed that current alcohol consumption followed a similar pattern as frequency of alcohol consumption (all *p* < 0.05), while former alcohol drinking pictured a reversed association with the health status metric only among European and African regions (all *p* < 0.01).

**Figure 3:**
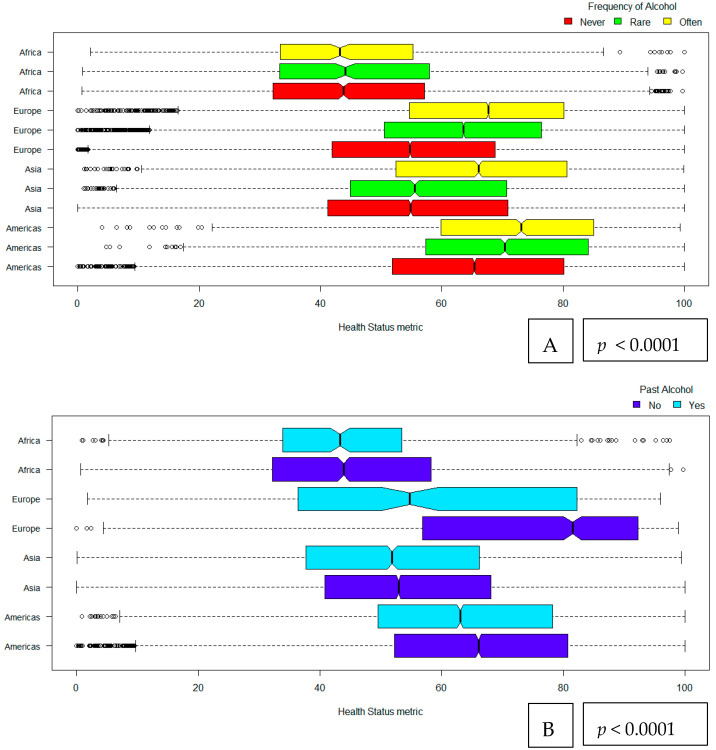

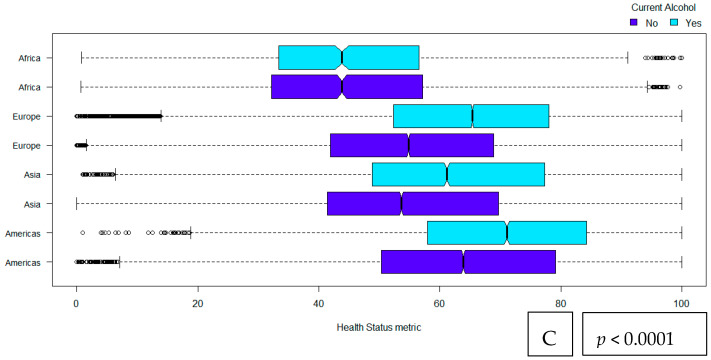
Alcohol drinking [frequency of drinking (**A**), past drinking (**B**) and current drinking (**C**)] and its relationship with health status metric among older adults by super-regions. Frequency of alcohol drinking is expressed as never, rare and often. Past and current alcohol drinking are following a binary categorization of yes vs no. Americas includes the regions of North and South America; Asia includes regions in Asia and Oceania as they are reflected within the ATHLOS data.

Alcohol drinking (evaluated as the frequency of alcohol intake, past and current alcohol drinking) and its relationship with health status in ageing by super-regions is visualised in Figure 3. Among all super-regions (Asia and Oceania, Europe, Africa, North and South America) frequent alcohol drinking was related to better health status (*p* ≤ 0.001). Older adults that were frequent alcohol consumers and were living in Europe and North and South America had the higher levels of the health status metric. Similar patterns are pictured for current and past alcohol drinking, respectively, with health status (all *p* < 0.0001).

## 4. Discussion

The present study revealed a high prevalence of current alcohol drinkers among older adults across the world; however, only one out of four reported a frequent alcohol consumption. The pooled adjusted analysis demonstrated a positive association between better health status and frequent alcohol consumption, while a similar relation transpired for the current drinkers. The association between alcohol drinking and older adult’s health status was significant among the “younger” older adults and octogenarians, females and among different financial levels. Stratified analysis according to alcohol drinking pattern revealed that until the age group of 85–94 years old, often alcohol drinkers, followed by rare drinkers, had better health status, as compared to never drinkers. However, after the age group of 85–94 years, no differences on health status were observed between rare and often drinkers, whereas, never drinkers continued having the lowest health status score. When regional analysis applied, the former association, either by frequency or current alcohol intake, was particularly pronounced among North and South Americans as well as Europeans. These results point to the targeted public health actions such as recommendation of frequent alcohol drinking but in low amounts, in improving or maintaining a high health status among older adults, internationally.

The present study has a major strength. To the best of our knowledge, this is the first multi-continent study to evaluate the association between older adult’s health status and alcohol drinking, using 17 large ageing cohorts, including data from (both developing and developed) 38 countries, thus allowing for international comparison of different settings.

Some previous findings reported a protective effect of moderate or low alcohol intake in various health outcomes [4], whereas recent research suggested no protective effect of alcohol intake on CVD health and mortality [5,6]. These studies have strengths (i.e., sophisticated analysis such as mendelian randomisation) and limitations (i.e., small sample, confounding controlling); however, to date, only one study [14] had reported results on the group of older adults. Our results applying pooled ageing cohort’s analysis, reported that the actual often-level frequency alcohol drinking, of at least 1 drink/week, is related to better health status among older adults around the world. Knott et al. [9] reported a beneficial relation between alcohol drinking and all-cause mortality only among 65+ years old females residing in England. Additionally, our findings are in accordance with the studies in which worse health outcomes and an increased risk of death were found in former drinkers [21,22]. It must be noted that when age group analysis is applied, the beneficial effect of current or often alcohol consumption attenuated after the age group point of 85–94 years old (Figure 1 and Figure 2). Beyond the age of 95, past alcohol drinking or rare/no alcohol consumption appeared to be related to better health status. These findings could be explained through the metabolism turn down in advanced ageing. The resting metabolic rate and respiratory quotient is lower in the very old individuals compared with the younger ones [23]. Macronutrient oxidation is impacted among the older in comparison with younger ages [24]. Ethanol is metabolised in a slower pace, due to a lower liver metabolic rate, in advanced age in parallel with the effect of medication interactions [25]. These facts, together with alcohol’s ability to promote social engagement [26], the lack of global age-, gender-, regional- and financial- specific data, suggests that alcohol consumption may affect the older population’s health status during the ageing process. This should be taken in account in order to plan targeted and effective alcohol-related health policies.

Assessing the effect of alcohol consumption in health status across diverge sub-population groups revealed differences between alcohol drinking affects. Often alcohol drinking was beneficial for the females, among all regions except Africa, both “younger” older adults and the octogenarians, as well as among all financial levels. Results on current and former alcohol drinking reported similar trends; however, they were attenuated among the different older population sub-groups. The positive effect of alcohol intake between female elders have described before among English populations [9]; however, the rest of our results are described for the first time, through a multinational context, and could help to develop the limited body of evidence concerning specific sub-group associations between alcohol drinking and health status in older adults. Interestingly our results pictured the higher coefficient magnitude between often-level frequency of alcohol consumption and healthy status metric of North and South Americans as well as in Europeans and Asians. From the three alcohol drinking items analysed in this work, only former alcohol drinking was inversely related to health status metric among the residents of Africa and Europeans. Moreover, often alcohol consumption had a consistent beneficial magnitude pattern with health status among all income levels. The greater effect marked among the richest group, while the lower effect was shown among the middle- income levels. The current pattern could be attributed to the differences in alcohol drinking behaviours such as in the quality and quantity [27,28], that has described within different socio-economic statuses. Studies in the general population have shown that burden of disease and mortality due to alcohol consumption is increased among lower income levels [28,29]. The interrelated pathways between alcohol consumption, financial status and healthy ageing remain unclear due to limited information in the field and require further exploration. Thus, it is crucial for health policy makers and stakeholders to plan targeted alcohol drinking strategies among different older populations, considering advance age, gender, socio-economic status as well as the region of residence. Further studies, especially with more precise information on the consumed amount of alcohol, are needed to confirm our findings. From this perspective, alcohol consumption in low amounts has been reported as beneficial for CVD health and mortality, to date [30].

The previously discussed findings also point to the need to rethink alcohol consumption guidelines and recommendations in the context of healthy ageing, taking into account not only comorbidities but also functional and physical limitation states. Alcohol consumption has been found to be inversely related to physical and functional limitations among elders [31]. As others have remarked, there are almost no alcohol drinking policies for the older population [10]. The current analysis could work as a first guide to the age threshold over which alcohol drinking is not related to a healthy ageing state. Our results could serve as the first international research work to create a pathway for future targeted alcohol drinking recommendations and policies among older adults.

### Limitations

The fact that this is a pooled cross-sectional analysis limits the potential for etiological conclusions. In addition, self-reported alcohol consumption data, such as those used in this study, may be prone to report and recall bias [32]. Our analysis was adjusted for several confounders (i.e., smoking habits, established CVDs); however, we could not adjust for other psychiatric problems and drugs with contraindications to alcohol intake, and thus our findings may be altered. In addition, the definition of alcohol drinking without including the actual amount of intake, and the actual estimation of ethanol intake, further limits our results. However, this kind of detailed information is not available in the ATHLOS harmonised mega-dataset [16].

## 5. Conclusions

The present work analysed the relationship between alcohol drinking and health status during ageing, among older populations internationally, using the pooled ATHLOS mega-dataset. Being a current drinker and consuming alcohol more often than once a week was positively associated with a better health status assessed by health metrics based on the assessment of 41 self-reported characteristics related to impairments in bodily functions, limitations in daily living activities, cognitive and physical functioning. The association between often alcohol consumption and health status was significant for age groups below 80 years old and octogenarians, females and among different income levels. However, the differences in health by alcohol drinking status started to disappear over the age group of 85–94 years old. Favourable differences in health by drinking status were particularly pronounced in Europe and in America.

## Figures and Tables

**Table 1:** Baseline characteristics of the study—overall harmonised sample by alcohol drinking.

		Overall Harmonised *n*, (%)	Alcohol Drinking Frequency				Current Drinker			Past Drinker		
Characteristic	Category		Never *n*, (%)	Rare *n*, (%)	Often *n*, (%)	*p* value	No *n*, (%)	Yes *n*, (%)	*p* value	No *n*, (%)	Yes *n*, (%)	*p* value
Sex	Female	75,246 (55.56%)	41,533 (33.70%)	17,594 (14.27%)	8518 (6.91%)	*p* < 0.001	45,507 (34.93%)	26,689 (20.48%)	*p* < 0.001	20,736 (51.79%)	4019 (10.03%)	*p* < 0.001
	Male	60,194 (44.44%)	21,060 (17.09%)	16,020 (12.99%)	18,528 (15.03%)		22,900 (17.58%)	35,158 (26.99%)		8809 (22.00%)	6473 (16.16%)	
Education	≤Primary	25,131 (20.31%)	14,235 (12.59%)	4045 (3.58%)	3323 (2.94%)	*p* < 0.001	16,784 (13.95%)	7584 (6.30%)	*p* < 0.001	10,758 (30.26%)	4138 (11.64%)	*p* < 0.001
	Primary	36,910 (29.83%)	18,581 (16.43%)	7433 (6.57%)	6365 (5.63%)		21,279 (17.69%)	14,275 (11.86%)		7133 (20.06%)	3587 (10.09%)	
	Secondary	45,673 (36.92%)	18,665 (16.50%)	14,534 (12.85%)	10,504 (9.29%)		19,265 (16.01%)	25,427 (2.11%)		5258 (14.79%)	1793 (5.04%)	
	Tertiary	16,005 (12.94%)	5329 (4.71%)	5175 (4.57%)	4907 (4.34%)		5458 (4.53%)	10,205 (8.48%)		2275 (6.39%)	605 (1.70%)	
Wealth	1st Quintile	30,583 (25.52%)	16,590 (15.05%)	6439 (5.84%)	4345 (3.94%)	*p* < 0.001	18,207 (15.68%)	11,114 (9.57%)	*p* < 0.001	7721 (21.37%)	2539 (7.02%)	*p* < 0.001
	2nd Quintile	26,753 (22.32%)	12,421 (11.27%)	7006 (6.35%)	5279 (4.79%)		13,441 (11.57%)	12,545 (10.80%)		4423 (12.24%)	1434 (3.96%)	
	3rd Quintile	24,928 (20.80%)	11,819 (10.72%)	6392 (5.80%)	4953 (4.49%)		12,653 (10.89%)	11,602 (9.99%)		5652 (15.64%)	1874 (5.18%)	
	4th Quintile	19,977 (16.67%)	9053 (8.21%)	5242 (4.75%)	4334 (3.93%)		9617 (8.28%)	9818 (8.45%)		4494 (12.44%)	1729 (4.78%)	
	5th Quintile	17,597 (14.68%)	7793 (7.07%)	4273 (3.87%)	4226 (3.83%)		8341 (7.18%)	8755 (7.54%)		4376 (12.11%)	1880 (5.20%)	
Ever smoker	Yes	55,383 (41.79%)	19,379 (15.76%)	15,875 (12.91%)	16,866 (13.71%)	*p* < 0.001	20,863 (16.02%)	33,478 (25.71%)	*p* < 0.001	7268 (18.05%)	5870 (14.58%)	*p* < 0.001
Physical activity	High	6812 (24.73%)	3912 (15.2%)	1203 (4.68%)	1321 (5.14%)	*p* < 0.001	3896 (15.15%)	2554 (9.93%)	*p* < 0.001	2710 (16.40%)	1011 (6.12%)	*p* < 0.001
	Moderate	9864 (35.81%)	6575 (25.61%)	1422 (5.54%)	1291 (5.03%)		6604 (26.68%)	2690 (10.46%)		5371 (32.51%)	1624 (9.83%)	
	Low	6799 ((24.68%)	4493 (17.50%)	1090 (4.24%)	808 (3.14%)		4541 (17.66%)	1851 (7.19%)		3353 (20.29%)	995 (6.02%)	
	Inactive	4070 (14.77%)	2330 (9.07%)	490 (1.90%)	729 (2.84%)		2298 (8.93%)	1279 (4.97%)		1153 (6.97%)	302 (1.82%)	
CVD	Yes	22,056 (20.15%)	10,497 (10.24%)	6376 (6.22%)	4194 (4.09%)	*p* < 0.001	10,820 (10.15%)	10,614 (9.95%)	*p* = 0.93	2897 (8.95%)	1282 (3.96%)	*p* < 0.001

Ageing Trajectories of Health: Longitudinal Opportunities and Synergies (ATHLOS) project; education classification was based on the International Standard Classification of Education; physical activity’s four classification groups were created based on the frequency and intensity of physical activity and inactive group reflects no physical activity.

**Table 2:** Results from multiple linear regression models that evaluated the association between healthy ageing and the alcohol drinking, among the *n* = 135,440 ATHLOS study participants.

Outcome	Categories	b-Coefficient	95%CI
Model 1			
Frequency of alcohol drinking	*Reference*		
	Rare	−0.50	−1.43, 0.43
	Often	3.70 ***	2.67, 4.73
Model 2			
Current alcohol drinking	Yes vs. No	1.32 **	0.45, 2.19
Model 3			
Past alcohol drinking	Yes vs. No	−0.83 *	−1.51, −0.16

Ageing Trajectories of Health: Longitudinal Opportunities and Synergies (ATHLOS); models are mutually adjusted for age, sex, body mass index (BMI), financial and education status, living alone, smoking habits, physical activity and established CVD. Model 1: R^2^ = 0.34, Model 2: R^2^ = 0.33, Model 3: R^2^ = 0.33; * *p* < 0.01, ** *p* < 0.001, *** *p* < 0.0001.

**Table 3:** Pooled estimates of the association between healthy ageing and the alcohol intake estimated in the overall sample, by age group, by gender, by wealth status and by super-region.

Outcome	Categories	b-Coefficient (±SE), R^2^												
		65–80	80+	Males	Females	Poorest	Poorer	Middle	Richer	Richest	Europe	N. and S. America	Asia and Oceania	Africa
	**Model 1**													
Frequency of alcohol drinking	*Reference*													
	Rare	4.96 *** (±0.18)	3.84 *** (±0.43)	−1.49 (±0.78)	−0.19 (±0.60)	−0.36 (±0.82)	0.20 (±0.84)	−0.87 (±1.13)	−2.53 ^#^ (±1.45)	−0.20 (±2.02)	4.22 *** (±0.17)	1.21 (±1.63)	2.25 ** (±0.83)	−1.66 (±2.55)
	Often	6.30 *** (±0.19), R^2^ = 0.23	6.63 *** (±0.48), R^2^ = 0.19	1.10 (±0.81), R^2^ = 0.30	5.62 *** (±0.71), R^2^ = 0.36	3.40 *** (±0.99), R^2^ = 0.32	4.06 *** (±0.94), R^2^ = 0.32	2.50 * (±1.20), R^2^ = 0.34	2.96 ^#^ (±1.51), R^2^ = 0.36	4.70 * (±1.86), R^2^ = 0.32	5.90 *** (±0.18), R^2^ = 0.30	13.01 *** (±3.46), R^2^ = 0.20	6.09 *** (±1.06), R^2^ = 0.19	0.14 (±3.43), R^2^ = 0.08
	**Model 2**													
Current alcohol drinking	Yes vs. No	5.53 *** (±0.16), R^2^ = 0.23	4.97 *** (±0.37), R^2^ = 0.19	−0.38 (±0.73), R^2^ = 0.30	2.13 *** (±0.55), R^2^ = 0.35	0.93 (±0.75). R^2^ = 0.32	1.90 * (±0.77), R^2^ = 0.32	0.76 (±1.05), R^2^ = 0.33	0.11 (±1.40), R^2^ = 0.34	3.41 ^#^ (±1.79). R^2^ = 0.31	1.32 ** (±0.44), R^2^ = 0.33	2.87 ^#^(±1.55), R^2^ = 0.19	3.59 *** (±0.79), R^2^ = 0.19	−1.11 (±2.29), R^2^ = 0.08
	**Model 3**													
Past alcohol drinking	Yes vs. No	−0.92 (±1.11), R^2^ = 0.09	−8.47 ** (±3.10), R^2^ = 0.18	−0.71 (±1.39), R^2^ = 0.10	−3.86 * (±1.61), R^2^ = 0.15	−3.46 (±2.20), R^2^ = 0.13	−5.75 ** (±2.20), R^2^ = 0.15	1.68 (±2.45), R^2^ = 0.14	0.80 (±2.38), R^2^ = 0.14	−1.89 (±2.55), R^2^ = 0.12	−6.62 *** (±1.58), R^2^ = 0.28	0.54 (±1.73), R^2^ = 0.20	−0.76 (±1.46), R^2^ = 0.10	−7.72 * (±3.42), R^2^ = 0.10

Models are mutually adjusted for age, sex, BMI, financial and education status, living alone, smoking habits, physical activity and established CVD. ^#^ < 0.05, * *p* < 0.01, ** *p* < 0.001, *** *p* < 0.0001. N. and S. America: North and South America.

## Data Availability

Data of the ATHLOS data are available upon request. The corresponding author (T.S.) affirms that the manuscript is an honest, accurate, and transparent account of the study being reported; that no important aspects of the study have been omitted; and that any discrepancies from the study as planned (and, if relevant, registered) have been explained.
